# A systems perspective on the importance of global health strategy developments for accomplishing today’s Sustainable Development Goals

**DOI:** 10.1093/heapol/czz042

**Published:** 2019-07-30

**Authors:** Jens Byskov, Stephen Maluka, Bruno Marchal, Elizabeth H Shayo, Astrid Blystad, Salome Bukachi, Joseph M Zulu, Charles Michelo, Anna-Karin Hurtig, Paul Bloch

**Affiliations:** 1 Research and Health Systems Advisor, School of Public Health, Ridgeway Campus, University of Zambia, Lusaka, Zambia; 2 Institute of Development Studies, University of Dar Es Salaam, Tanzania; 3 Department of Public Health, Institute of Tropical Medicine, Antwerp, Nationalestraat 155, B Antwerpen, Belgium; 4 National Institute for Medical Research (NIMR), Dar Es Salaam, Tanzania; 5 Department of Global Health and Primary Care, University of Bergen, Norway; 6 Institute of Anthropology, Gender and African Studies University of Nairobi, Nairobi, Kenya; 7 School of Public Health, Ridgeway Campus, University of Zambia, Lusaka, Zambia; 9 Department of Epidemiology and Global Health, Umeå University, SE, Umea, Sweden; 10 Steno Diabetes Center Copenhagen, Niels Steensens Vej 6, DK Gentofte, Denmark

**Keywords:** Accountability, democracy, determinants, developing countries, ethics, health systems, organizational change, outcomes, participation, priority setting

## Abstract

Priority setting within health systems has not led to accountable, fair and sustainable solutions to improving population health. Providers, users and other stakeholders each have their own health and service priorities based on selected evidence, own values, expertise and preferences. Based on a historical account, this article analyses if contemporary health systems are appropriate to optimize population health within the framework of cross cutting targets of the Sustainable Development Goals (SDGs). We applied a scoping review approach to identify and review literature of scientific databases and other programmatic web and library-based documents on historical and contemporary health systems policies and strategies at the global level. Early literature supported the 1977 launching of the global target of Health for All by the year 2000. Reviewed literature was used to provide a historical overview of systems components of global health strategies through describing the conceptualizations of health determinants, user involvement and mechanisms of priority setting over time, and analysing the importance of historical developments on barriers and opportunities to accomplish the SDGs. Definitions, scope and application of health systems-associated priority setting fluctuated and main health determinants and user influence on global health systems and priority setting remained limited. In exploring reasons for the identified lack of SDG-associated health systems and priority setting processes, we discuss issues of accountability, vested interests, ethics and democratic legitimacy as conditional for future sustainability of population health. To accomplish the SDGs health systems must engage beyond their own sector boundary. New approaches to Health in All Policies and One Health may be conducive for scaling up more democratic and inclusive priority setting processes based on proper process guidelines from successful pilots. Sustainable development depends on population preferences supported by technical and managerial expertise.

**Key Messages**

- The dominant reliance on evidence from technical innovations and from expertise in priority setting has not yet lead to either adequate health coverage, nor to health systems that can achieve sustainable solutions to improve population health.
- A historical account of health strategy and systems developments till today showed that health systems definitions and practice remain insufficient to address population health determinant associated targets within most of the Sustainable Development Goals (SDGs).
- We propose a strong focus on accountability and democratic legitimacy within health systems and SDGs as conditional for future sustainability of population health improvements

## Introduction

The ever-increasing evidence and technical developments supporting population health has not yet reached the goal of Health for All, which was set for year 2000 as determined by the World Health Assembly in 1977 (WHO, 1977).

**Figure 1:**
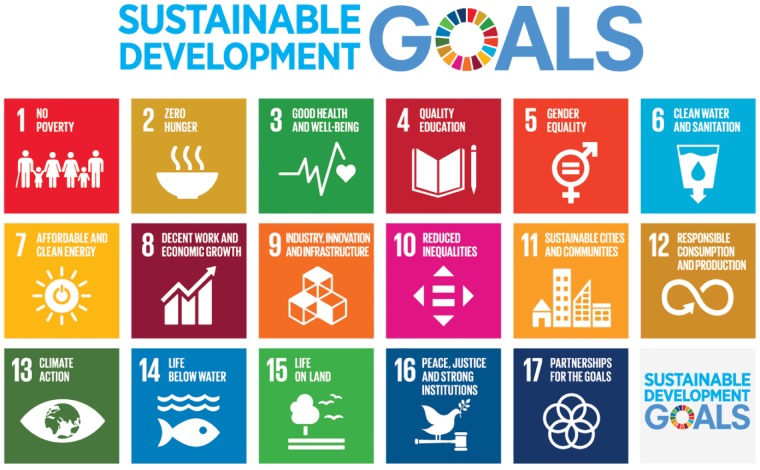
Sustainable Development Goals (United Nations, 2016). Approved by UNDP for non UN organizations.

The decision-making for population health has not led to optimally accountable, fair and sustainable solutions. The health sector remains globally marred by inequities, intervention inadequacies and gaps in coverage. Technical experts, politicians, managers, service providers, community members and beneficiaries each have their own values, expertise and preferences, to be considered for necessary buy-in and sustainability. A 2015 paper raised concerns on limited stakeholder inclusiveness in current approaches to Health Technology Assessments (HTA) (Daniels *et al.*, 2015) and found that such inclusiveness could provide opportunity for democratic learning. That argument prompted a commentary, whether the same concern also applied to overall priority setting and planning in health systems (Byskov *et al.*, 2016) and raised whether the Sustainable Development Goals (SDG) (United Nations, 2015a) could provide added reasons for more inclusive and participatory approaches (Figure 1).

Determinants of health are embedded within targets of most of the SDGs. In the Annexe, a health systems associated target is exemplified under each of the 17 goals.

The SDGs were formulated through comprehensive consultative processes including multiple sectors and civil society. Their degree of global population-based legitimacy is therefore higher than in the Millennium Development Goals (MDG) (United Nations, 2015b) and other global initiatives not based on similar processes.

Determinants of health were also prominent in the Health for All decision of 1977 (WHO, 1977): From its executive summary: ‘as a minimum, all people in all countries should have at least such a level of health that they are capable of working productively and of participating actively in the social life of the community in which they live’.

The Health for All goal is now again and with reference to the SDGs coming into focus in WHO strategy developments.

On that basis, this article addresses the following research question:

To what extent are contemporary health systems and their processes of priority setting and user involvement capable of optimizing population health and how may they contribute to accomplish the health determinant targets within most of the SDGs?

We aim to support more accelerated population health development by drawing attention to democratic and motivational drivers of sustainable change processes that are less visible in the global health systems discourse.

## Methods

We applied a scoping review approach (Colquhoun *et al.*, 2014; Brainard and Hunter, 2016) to literature on health systems from PubMed, Google Scholar, web identifiable international organization and international programme reports and own institutionally based libraries.

First, we scanned the web by terms for global, international, bilateral and developing country health policies and strategies, health systems and health services. The most useful hits appeared by searching for *health systems strategies global.* Due to the importance of early literature that may have laid the conceptual ground for the Health for All goal from 1977 (WHO, 1977), we covered the last 50 year period (since 1968). That yielded 59 000 000 overall entries and was by limiting to scholarly publications reduced to 408 000. However, browsing through the entries did show the wide range of types of documents from research, plans, communications, etc. It also showed that strategy implementing organization not surprisingly tended to promote their own approach. In addition, the distinction between health systems and health services has remained unclear with increasing use of the now more popular systems term for services and service management. We explored several searches until main features had appeared and repeated themselves without clear additional ones coming up.

For peer-reviewed literature, we mainly relied on PubMed Medline searches on health systems and the terms theory, systems methods and analyses in various and/or combinations. We looked for those that in abstracts included a balanced provider and user inclusive scope and a main but not exclusive focus on low- and middle-income countries. The most relevant output of 1223 papers emerged from a search on health systems theory, methods and analysis since 1970.

Going into abstracts and actual papers of highest relevance led to a number of papers including reviews, scoping reviews, complex adaptive systems, theories of change, realist approaches and evaluations. We excluded those that by their titles had a specific disease, or very confined service programme focus. Indications of a possibly broader health systems relevance or a developing country focus led to abstract reading of 60 of them and full text reading of 20. They spanned the above-mentioned methods and largely outlined needs for complex methodological approaches and new interventions, but without solutions or ensured scale up of their priority setting, funding and management. The complexity of labelling, definitions, methodologies, scope and reporting is a finding in itself and did not provide adequate references to answer our health determinant focused study question.

Reference to the comprehensive systems issues that we raise do appear but are only given prominence in a few documents on which we base definitions and analyses.

A comprehensive health systems definition emerges from a WHO report by Schaefer’s (1974) *Administration of Environmental Health Programs—A systems view*. It represents a highly coherent and practical application-oriented reference for health systems thinking and analyses. The inherent principles for comprehensive health systems were further elaborated in a report of a WHO Expert Committee on application of systems analysis to health management in World Health Organization, Technical Report Series, No. 596, 1976 (Report of a WHO Expert Committee, 1976). The technical report (Report of a WHO Expert Committee, 1976, p. 7) defines health as the result of occurrences in many sectors or the social system, which may support, constrain or even counteract desired results of interventions in the health sector. This expands the scope of health management from service provision only to improvement action for community health by all available means.

To us these early documents are defining for a comprehensive health systems definition which has not been expressed with similar systems stringency since.

**Figure 2:**
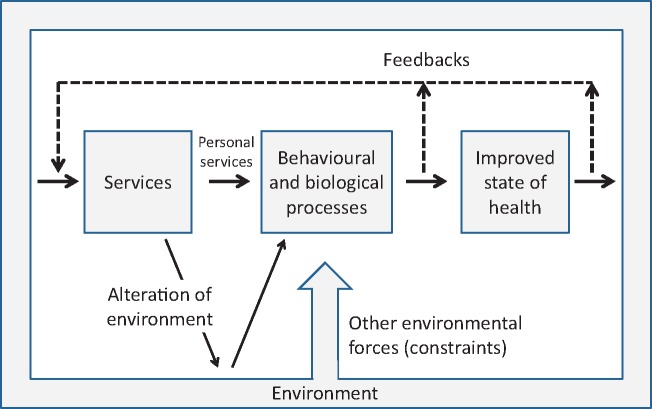
Environmental, social and service determinants for states of health. Figure reproduced from Schaefer (1974, p. 95) with permission from WHO).

Schaefer illustrates this in a summarizing figure of his report (Figure 2).

This figure presents a model or framework for assessment towards health systems models appearing in numerous subsequent systems approaches. To align with more current terminology, services should rather read interventions and the environment covers both physical and social determinants and contexts.

The figure is multidimensional in capturing not only the basic planning cycle, but also key other parallel processes and ‘forces’ that are pertinent in priority setting decisions for achieving health and development. It refers to multi-stakeholder and community involvement, which are now again receiving increased attention.

We define health systems as to comprise a broad cross sector, determinant and civil society inclusive health systems concept. The health sector is included as a sub-system and the health services, disease- and programme-specific interventions are mostly subsystems within the health sector. The distinction is important as each such defined system requires specific aims, capacities, management and financing. Without clarity on specific systems boundaries, their structures and processes may not lead to feasible and indeed sustainable outcomes.

We identified and listed well-described health systems strategies at national and international levels from their introduction in books, plans and reports till date of writing this article. We attempt a sequential summary presentation based on their inception, though some continued to be applied in parallel. For each systems heading, we indicate a year of inception and illustrate the systems focus by a possible concurrent broader development system. Among references, we include associated analyses and illustrate critical views. We are aware that a multitude of programmes and approaches exist which are aligned with particular national political systems, particular international non-governmental or sub-national civil society organizations. These provide many non-cohesive model, mechanisms and showed local achievements, but were not scaled up or continued.

## Results

The findings are presented in progressive time sequence according to main characteristics of the health systems over time. There are some overlaps in health systems priority setting characteristics from time to time and also more continuous parallel existence. We were able to identify stages of Health Systems definitions and developments from the late colonial period in the 1950s and to date. Each of them may be analysed in much greater detail, but for the aim of an overview in this article, we focus on summaries and assessments of their main features.

### Hospital services and hygiene-focused public health: ‘up to 1960—colonial period’

Medical and health services globally and in the colonial period mainly prioritized hospitals and added efforts to improve prevention of mainly hygiene associated diseases and outbreaks through strict Public Health regulations and practice by authorities. Coverage and equity remained as serious problems, but it was assumed that higher level service improvements and nationally enforced Public Health programmes would eventually benefit health of populations. Some ‘trickle down’ benefit could surely be expected and has continued as an element of later health systems developments, but has also been considered as insufficient for achieving population health (Jafar, 2015). The stringent colonial management surely achieved results within its defined objectives and more balanced approaches directly supporting both service provision and population health indicators have been accounted for as well in a case from Ceylon at that time (Jones, 2002).

### Efforts at better coverage and coherent systems: ‘1960s—new states’

A policy and strategic basis for developing a national systems approach to health, especially in developing countries was provided in the book ‘Medical Care in Developing Countries’ (King, 1966). It argued for a change from earlier hospital service and hygiene control-oriented Public Health to a better coverage by first contact facilities and services and more appropriate hospital services. A further perspective is the co-development of national health services in the continued presence of NGOs from the colonial era in Tanzania (Jennings, 2015). It illustrates a case of transition and both advantages and difficulties from existing competence and resources of colonial area structures, which in new countries diversify and include aid agencies while now national priorities also claim influence.

The national health systems approaches and priority setting detail were also shaped by the different political alignments with politically based priority setting agendas of the so-called East and West development agendas, respectively.

A summary from Vietnam (Pruzin, 1996) may illustrate such differences. This case illustrates how a communist developing country with an equity and low resource health sector accommodates medical advances and economic growth in a more market-oriented economy, though also raising questions about coverage and access to advances. We have within the scope of this article not expanded with comparable references on Russia and countries previously under the Soviet Union.

Such structural changes have continued in parallel with, the overview summaries of this article, but do not contradict them.

### Systems development for health: ‘1970s—new governance’

The comprehensive WHO report by Schaefer’s (1974)*Administration of Environmental Health Programs—A systems view* probably still stands as the most relevant reference for practical application of later health systems thinking and analyses.

The 1976 report of a WHO Expert Committee on application of systems analysis to health management is a further consolidation of standards for health systems thinking. These two WHO documents (Schaefer, 1974; Report of a WHO Expert Committee, 1976) have already been referred to in the methodology section as they were realized to represent the most comprehensive systems theory and practice as a reference standard for this article.

### A health systems consensus: ‘late 1970s—global co-ordination’

The 1978 *Primary Health Care* (PHC) approach. PHC was globally endorsed (and never replaced) and was further described and assessed in 1990 (Streefland and Chabot, 1990, McPake *et al.*, 1993). The five key principles of the approach were: Equity, focus on Prevention, Appropriate Technology, Inter-sectorial Collaboration and Community Participation. Health for all and not only health care for all was the overall aim.

Contrary to the word primary, though that was a prioritized aspect at the time, the principles were to be applied to priority setting within whole health systems including national level specialist services in a way that ideally provided equal chance for any citizen to receive the most advanced treatment in the country if needed and available.

Managerial and economic aspects were less explicit, but the Appropriate Technology principle does include some cost-effectiveness thinking, though still related to the overall equity principle. A strength was that ownership of health agendas was transferred to individual developing countries. The PHC principles were also maintained in many national strategic documents as a way of retaining options for locally customized and owned solutions.

### Donor dominance, programmatic fragmentation and supply-based approaches: ‘1980s—structural adjustments’

However, local customizations made national programmes more difficult to manage and budget for by international agencies and bilateral donors. Within the first few years after its creation fragmentation of the holistic PHC approach started by selective PHC approaches which were by international organizations and bilateral donors considered necessary to persuade donor countries to provide the funds for implementing support programmes (Magnussen *et al.*, 2004). This disempowered the counties in priority setting by contradicting the coherent PHC concept, turning it into primary level contact only, or into more separate programmatic approaches that guided rather complete globally pre-determined programmes. Some consequences are illustrated from such transition in Peru (Menon, 2007). This country case represents that reproductive health as a core aspect of population health is very equity dependent, and that medical technology and health sector quality advances may be counterproductive for reproductive health averages due to poverty level increases in spite of economic growth.

These developments are best described as a distancing from a comprehensive systems approach to more supply-oriented health sector elements.

### Burden of disease and programmatic efficiency: ‘1990s—conditions for development support’

Economic crises led to a request for much more stringent economic principles based and pre-determined planning. This was exemplified in the World Development Report (The World Bank, 1993). There was a refocus on cost effectiveness of specific services and output rather than on context, processes and participation. A strong focus on privatization characterized some developments, which provided added opportunities for mainly the better resourced part of the population.

The coherent priority setting at sub-national and national levels was further weakened. In this scenario, there was increasing understanding of the necessity of Sector Wide Approaches (SWAp) (Hutton and Tanner, 2004), country ownership and donor co-ordination, but approaches remained largely prescriptive by aid agencies by being tied to their poorly co-ordinated conditions for recipients to comply with.

Disability Adjusted Life Years (DALY) (The World Bank, 1993) became helpful tools that are still being used, but do represent compound evidence-based indicators that rest on a number of assumptions and basic standardizations. These are not easy to fully understand and accept as valid tools nationally and particularly not at sub-national levels. DALYs provided support for approaches that allowed a focus on cost effectiveness of specific services and output and in the process led to lesser focus on context, processes and participation.

National health policies and plans were developed based on the DALY measures, survey based and routine health information systems data. With main priorities set at national level, thus conditioned detailed plans were to roll up from facility and district levels.

The focus on quality, efficiency, cost containment, cost effectiveness and performance became not only the main managerial guidance, but also resulted in financially and operationally conflicting priorities.

The importance of explicit and mutually agreed priority setting remained poorly supported and managed in the by health systems targeted district level realities (Tanner, 2005). Such continued examples include that commonly much less than the nationally defined budgets for district activities will materialize or be regularly disbursed (Maluka *et al.*, 2010). Any kind of initial priority setting breaks down in such situations during the planned for year and it becomes too haphazard what is done or not done.

### A multitude of approaches: ‘2000s—neoliberal dominance and new goals’

SWAp, DALY and similar managerial and evidence-based approaches (The World Bank, 1993; Hutton and Tanner, 2004) led to development and funding of programmes in a new global policy and funding scenario. They include amongst other, the Global Fund to Fight AIDS, Tuberculosis and Malaria (Global Fund) (Mounier-Jack *et al.*, 2010; Warren *et al.*, 2013). However, they mainly addressed infectious diseases of global importance, but their prioritization in relation to the disease focus in developing countries did not base on joint contextual assessment at country or sub-country levels (Maundy *et al.*, 2007). The mentioned global and other programmes recognized the importance of health systems, but the version promoted by each of them differed. They did not always respond to wider health needs and preferences of populations (Oluwole, 2008).

The MDG’s (United Nations, 2015b) became a global development agenda to further assist in addressing global development inequities. They created new awareness and provided a range of contributions to the stated aims. They were, however, much dependent on voluntary benevolence of more resourceful populations, nations and organizations. Concepts and solutions remained mainly based on burden of disease and programmatic efficiency concepts.

### Goals, values, ethics and fairness in priority setting: ‘2000s—new social and development models’

Values and ethics were prominent in the PHC approach (Streefland and Chabot, 1990). Associated fair and democratically legitimate principles were again conceptualized in the Accountability for Reasonableness (AFR) approach (Daniels and Sabin, 1997; Martin and Singer, 2003). It represents a process focus for agreement between individuals and organizations representing all relevant partners from all levels and not least the service users though the best possible approximation to the four conditions: Relevance, Publicity, Appeals and joint Enforcement of first three conditions. The need for more people centred approaches (Biehl and Petryna, 2013) and more qualitative and participatory actions (Marmot, 2005; Greenhalgh *et al.*, 2016) has been argued by others, but have to date not been brought to scale in national and international health systems. The greater cross sector and social determinant focus (Marmot, 2005) also came up front in the health policy and systems publications and debates, but did not result in any major change within health systems. The potential for democratic learning through AFR-guided processes in HTA has already been raised (Daniels *et al.*, 2015).

### Systems thinking and universal health coverage: ‘late 2000s—setting new agendas for populations’

The managerial and dominantly provision focused health sector approaches were again brought in by the WHO publication on Systems Thinking. Each programme is based on resources which may weaken other programmes if their combined need for managerial resources is not respected. Six building blocks were defined (Alliance for Health Policy and Systems Research and World Health Organization, 2009, p. 31; Peters, 2014) as: (1) Health Service delivery, (2) Health workforce, (3) Health information system, (4) Health systems management, (5) Financing and (6) Leadership.

The requirements within each building block were comprehensively described ideal standards. The need for prioritizing between them for limited resources was poorly addressed. Their focus on service provision has been subject to critique (Mounier-Jack *et al.*, 2014; Lazarus and France, 2015).

Universal Health Coverage (UHC) (WHO, 2013) is guided by the Health Systems Thinking (Alliance for Health Policy and Systems Research and World Health Organization, 2009) and in our definition really represents quite strong versions of a mainly health sector and health service provision focus. It may also be an effort to ensure better management of the MDG-based programmes (United Nations, 2015b). Ethical and participatory aspects are considered, but this is not different from the often little concrete reference to such in purely health service-oriented programmes. The debate on stronger user involvement in UHC is ongoing (Norheim, 2015).

### Health in the SDGs: ‘2015 —global sustainability’

**Figure 3.**
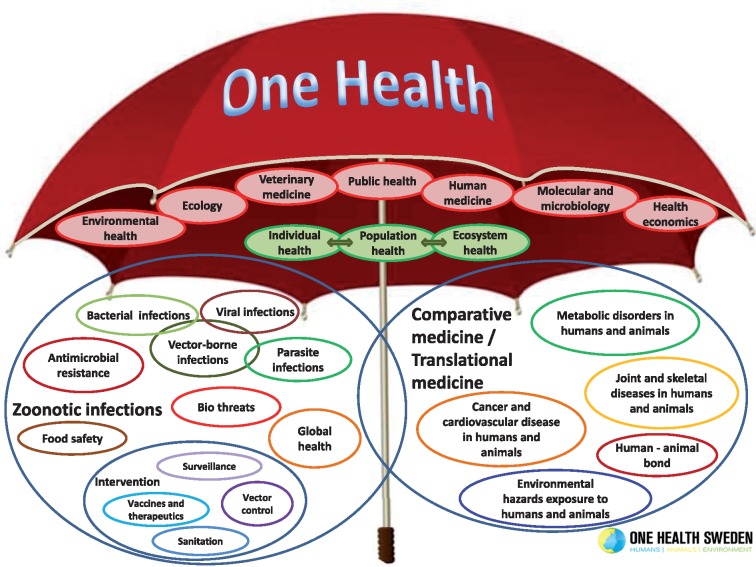
One Health. With permission from SLU Sweden (Lerner *et al.*, 2015).

Based on successes and failures of Millennium Development Goals (United Nations, 2015b) and the added insight from new social models, a health determinant focus was reinforced by the WHO initiated approach to include Health in All Policies (HiAP) (WHO, 2014) Additionally a One Health Approach (Global Risk Forum, 2015) for health of human, animals and the environment had been introduced. These two new approaches are both embedded in SDG’s (United Nations, 2015a) which also provide a new global context for the health sector. The principles of the SDGs correspond better to the health systems view of 1974 (Schaefer, 1974) than to later fragmentations and main service orientations. The One Health Approach (Global Risk Forum, 2015; Lerner *et al.*, 2015) is illustrative for necessary processes across all of the SDGs (Figure 3).

One Health was developed to control of Zoonotic diseases through co-ordinated interventions towards securing health of humans, animals and the environment and does match principles of SDGs for addressing health determinants within development. Healthy agricultural crops and sustainable environments are among such determinants for population health. A direct action on health determinants among most SDGs is suggested in a recent paper (Luyckx *et al.*, 2018) within a single specific programme. It shows how a more general action on determinants for a number of health and service problems may be minimized or can provide a better platform for effectiveness of specific approaches to prevention and care.

The current sustainability debate also explores the association of democracy and sustainability in relation to the environment, and is equally relevant for social systems including health. Illustrative dimensions of democracy, technology and values for environmental sustainability to associate with environmental sustainability have been explored by the Foundation for Democracy and Sustainable Development (Westall, 2015). Values must be respected in relation to scope of concerns and timeframes as well as to the level of technology in each setting. Types of democracies can be placed within such framework and their influence on sustainability be discussed.

The same dimensions are relevant within health systems concerning participation (Biehl and Petryna, 2013), but we shall only note the relevance of such debate, not try to structure it further in this article.

## Discussion

Fluctuating changes and inconsistencies in definitions, limitations of scope and application of health systems were identified through the historic account presented in this article. Their emergence seemed more political than based on standards for systems theory and practice. That questioned their relevance and validity for any consistency in efforts to improve population health which should be the key desired impact of any comprehensive health system. We have stated the need for health systems to focus on all SDGs, but to do so health systems also have to review their own internal priority setting processes. Some systems insight has been achieved in reference to newer concepts of complex adaptive systems (McDaniel *et al.*, 2009) and realist (Marchal *et al.*, 2012) approaches. These are important elaborations of what is already covered in the systems reference documents (Schaefer, 1974; Report of a WHO Expert Committee, 1976).

The results have provided new insight into the introductory questions and concerns. Within the previously defined health systems framework by WHO we shall therefore with reference to our results discuss important issues for health systems to optimally support achievement of not only health targets of SDG no. 3, but also health-related targets of other SDGs. In attempts to understand the identified lack of consistent health systems direction for sustainable population health, we refer to the value and process standards that guide priority setting within AFR. Health Systems development has surely not explicitly applied the four process guidance conditions of AFR (Daniels and Sabin, 1997; Martin and Singer, 2003). A necessary process guidance rests on an ethics-based approach to procedural fairness and legitimacy. The approach title includes accountability and reasonableness. We have found it feasible to organize the discussion of the findings in an AFR perspective under the issues of accountability, legitimacy and ethics. We then refer to the relevance for sustainability and reasons why we consider basic human relations and bottom up democratic practice, shared responsibility and shared monitoring as a necessary inclusion for sustainability of population health. More directive policy, economic theory, technical and past ‘hard’ evidence-based approaches have not proven sufficient.

### Accountability

Globally the national level democracies seem constrained and often inadequate because populations are not having experiences from permitted democratic practice of mutual accountability and associated principles for participation in priority setting at also sub-national levels (Pateman, 1970; Webler and Tuler, 2000; Biehl and Petryna, 2013; Westall, 2015).

National democracies base on individual voters. That principle has no requirement for knowledge, experience or competence in state governance among voters. In the health sector, provider and user knowledge, opinions and preferences on health are far better than for national governance.

The Paris Declaration of 2005 on donor co-operation and the associated Accra agenda for action of 2008 (OECD, 2005) can be considered as a constitution for mutual accountability in priority setting for development assistance. They are poorly complied with by aid agencies.

That lack of co-ordination from the dominant priority setting funding-based mechanisms led to fragmentation of the PHC (Magnussen *et al.*, 2004) globally agreed values and principles. The resulting national and international guidance became based on evidence and on a broad guidance from aims such as equity, quality and cost-effectiveness. Such aims are, however, contradictory and competitive within a fixed resource frame, and the necessary prioritization between them has not been transparent or accountable to recipients. A common argument of aid agencies in developing countries makes reference to programme acceptability to donor country taxpayers. That condition can stimulate nepotism and even corruption in recipient countries, raises concern on the same between agencies, and is an unacceptable cause of aid failures. The results have been serious degrees of programmatic wishful thinking, which is destructive for any coherent and sustainable health systems management at any level.

Concern has been raised on constraints from poorly controlled vested interests (Oluwole, 2008; Byskov *et al.*, 2016; Tanner, 2005). Personal and organizational self-interests will of course always exist, but must be accountable and not take non-transparent prominence in decision-making for aims to be reached and services to be provided. Health systems efforts are dominated by structures and establishments which to ensure own growth and sustenance are indeed highly ‘resilient’ to change and compromise. One can question if that consideration led to choice of global programmes that control infectious diseases that also represent an important risk to donor country populations and their frequent travel. Much evidence and effectiveness-based implementation programming attracts added resources and it is certainly expected that such extra input will lead towards desired results. However, if such resources are partly mobilized from current or intended resources for other programmes those may become less effective. A recent reference from the *Lancet* (Kruk *et al.*, 2018), argues that better quality health care supported in the SDG 3 framework will improve population health. It will, but not sufficiently and it will require unlimited resources and redistributions with the health sector prioritizing its own actions and growth above other sectors and overall SDG responsibilities for health determinants. The need for country-based policy formulation to achieve UHC at any resource level and to factor in monitoring of health impacting targets have also been stated by the Director General of WHO (Ghebreyesus, 2017).

Programmatic decisions are commonly based on results of effectiveness research, which has mostly only addressed resource needs and not resource availability. More resources are the mantra for added effectiveness, but that is not a justifiable request if not exposed to an adequate contextual assessment of operational feasibility and resource bases.

One factor for accountability of health systems development is the aid agency programming that makes extensive use of commercial consultancies towards heavily resource dependent and often managerially weak ministries. Such consultancies tend to neglect a time-consuming contextual alignment, or to accept Terms of Reference (TOR) which may be methodologically insufficient and directly biased by the external task giver. They are also not responsible for the decisions that they recommend. This is happening in a situation where both the national administration and the in-country aid agency representatives may be weak in analytical and methodological understanding. It is indeed anywhere in the world commonly seen that management avoids responsibility for defining necessary change, by directly implementing expert consultant recommendations without the necessary decision-making process in their context. However, national capabilities have increased and can deliver more contextually accountable advisory services to national authorities. Contracts are mostly awarded based on TORs and on fairly standard predetermined mainly organizational, administrative and technical criteria. They are to ensure objectivity, but that combination does not guarantee contextual relevance. Managers, not consultants or aid agency staff remain responsible for within country long term democratic accountability in application of results. Universities and associated sector research institutions can vet the quality of TORs and do a much more contextually valid analysis based on more appropriate TORs. Such units can do, e.g. appraisals, reviews and evaluations on a much more qualified basis partly due to their own assured quality conditions based on their accreditation. They may not compromise on validity of their approaches, methods and results if such tasks are integrated parts of their portfolios.

Popular accountability is also being promoted in networks such as Community of Practitioners on Accountability and Social Action in Health (COPASAH) (2019). Community skills in ensuring accountability is further supported by globally existing programmes for health literacy by governments and by networks such as Equinet Africa (2019).

### Legitimacy

Priority setting must be legitimate in terms of compliance with a combination of decision-making pre-conditions in legal and administrative frameworks, latest existing evidence on health, disease, intervention options and not least expressed preferences and values of both providers and users. The attention to values draws in the importance of fairness as agreement does not emerge from a purely logical rationalization of the pre-conditions. Concepts of fairness are important components of legitimacy, and appeared remarkably similar in selected countries (Byskov *et al.*, 2014). Agreement on fairness in our view bases more on involved stakeholders’ own summary assessment than on complex or standardized fairness criteria which may restrict the desired inclusion of all stakeholders. A guide for such process was developed and tested in the operational research project ‘Response to Accountable Priority Setting for Trust in Health Systems (REACT)’ (Byskov *et al.*, 2014). Similar lessons have emerged from other projects (Blas *et al.*, 2016), but larger scale up and evaluations have so far not been carried out.

Basic compliance and respect towards democratic principles (Pateman, 1970; Webler and Tuler, 2000) is virtually absent in most of the inconsistencies that we have documented. As it was seen during PHC fragmentation and during the efforts to address the SDGs, some organizations are trying to carve out space for their own agenda and funding without recognizing the need to balance that with other needs as required within the SDG agenda. Picking on supply aspects and promotion of own sector funding as for UHC is important, but must be balanced against the efforts to address wider determinants for population health. It is also of concern that the three sectors primarily involved in the One Health Approach for some years seem to not yet have accepted to become less concerned with who leads the activities than to jointly achieve the associated desired SDG targets.

### Ethics

In priority setting and involvement of the population the ethical choice must be clearer in relation to individual treatment and population health action outcomes. These partly represent a continuum, but each is an ethical imperative in its own right. The individual requirement to receive the currently most effective available treatment is an ethical imperative and a right, but so is the overall and equitable action for population health (WHO, 2014). It is rarely clarified in the debate that provision of more resources to one at the expense of the other is a value-based and political responsibility and cannot base on rational managerial or technical arguments only (Kisoka *et al.*, 2017; Tetui *et al.*, 2017).

Curative medicine will probably remain the most costly priority for the health sector, but allocations to population health should not be competed out by potentially unlimited treatment costs. Better population health will decrease many primary and repeat treatment and intervention costs and may make specific services and interventions better targeted to a remaining need. Consensus on the necessity of an ethical guidance as an imperative for population health will assist in simplifying choices in much health systems priority setting.

### Sustainability

The first three cross cutting issues led towards conditions for sustainability. The debate on defining and operationalizing more sustainable systems approaches by more strongly including a priority setting and a decision-making process guidance raises the question whether (1) technical evidence-based information is most important and can be improved by more participatory value and specific context-based approaches (Baltussen *et al.*, 2013) or (2) the participatory democratically based approaches (Biehl and Petryna, 2013; Daniels *et al.*, 2015) are most important, but need support from technical evidence. Based on findings in this article both are justified in their own right, but for reasons of democratic legitimacy and sustainability we need balanced solutions with participatory approaches being informed and not overruled by the technical ones. This dilemma is explored in the general sustainability and democratic governance debate (Westall, 2015).

The referred to commentary (Byskov *et al.*, 2016) questioned why AFR as an already developed and piloted (Byskov *et al.*, 2014) inclusive process for priority setting had not yet been scaled up. We also suggested that AFR might be easier to accept and operationalize if further explained as Accountability for Fairness and Rights for democratic process guidance within the SDGs. It is applicable in most organizational situations for facilitating joint priority setting decisions such as between ministries, between different portfolios within a Ministry of Health, between different professionals in a District Health Team, between civil society organizations and between community individuals. Existing advisory user panels, institutional boards and community consultations gain new importance in such processes. A challenge to power is often raised as a constraint, but the principle can on the contrary be seen to strengthen legitimate leadership for now improved decision-making, subsequent effective implementations and not least continued jointly owned monitoring and evaluation. Such must also identify if and when decisions may have to be reviewed. Democracy is also about letting go of decisions-making while ensuring the necessary clarity of more joint priority setting responsibilities. That is associated with the pains of devolution, which is pre-determined to fail if not including the full responsibility, the necessary authority and the associated funding. This is not easy, but requires an agreed process guidance such as AFR.

In face of increasing global environmental degradation and increasing mortal human conflict a more mutually accountable and more democratic national and global governance is urgently needed. Where did we forget the global ethics of human relations embedded in basic concepts of health, democracy, culture and religion? Have we become more inhuman than our forebears, the hunter gatherers who in small groups with mutual responsibility managed to conquer the world. Our personal mindsets have hardly changed since then and personal concepts of accountability and fairness remain globally very similar (Braithwaite, 2005; Byskov *et al.*, 2014). Do we now accept that competitive larger group identities and conflicting high level political and economic paradigms risk to destroy it? Already shared interpersonal face-to-face contact values need to dominate the global agenda. Most recently a publication in the Bulletin of WHO 2018 (Packard, 2018) has shown that the described situations in this article are well-known by others, but have so far not been adequately responded to. Change has to start somewhere and can emerge from SDGs and this proposed population health focus. The urgency is the already missed opportunities for sustainability. Some might argue that strongly autocratic states might more effectively force changes for population health. However, that is not an option for states that define themselves a democratic.

Sustainable global health depends on health of the globe as amongst other shown in a paper from 1990 (King, 1990) by the editor for the first health systems origins (King, 1966). There is a need for rethinking health sector and health systems responsibilities in measuring health outcomes and impact of health determinants across most SDGs. At least added insight is needed on optimizing sustainable population health impact from addressing such determinants. That calls for an increased focus on health systems priority setting processes rather than on specific disease, service, managerial and economic disease or programme targets.

## Conclusions

Today’s health systems have not responded to the fact that population health is mainly influenced by determinants outside the provider and service defined health sector. Therefore contemporary health systems are not appropriate for optimizing population health within the framework of all the SDGs.

## Suggested way forward

To support practical learning approaches towards achieving population health within the SDGs we propose to:

- Strengthen the One Health approach for human beings, animals and the environment.
- Address the environmental and social determinants for health through sectors, decentralized governance and civil society.
- Ensure transparency on vested interests and separation of those negotiable or not negotiable in a priority setting process.
- Strengthen sub-national democratic priority setting for health action by a major scale up of participatory approaches supported by mutually accountable decision-making process guidance such as from AFR.
- Define health-related process, outcome and impact benchmarking indicators which can monitor progress towards attainment of all SDGs.
- Evaluations and research for further in-depth understanding of attainments and constraints.

## Acknowledgements

The Dean and staff of the School of Public Health of the University of Zambia for professional support and advice. Colleagues at the Faculty of Health and Medical Sciences of the University of Copenhagen for supplementing insight on One Health, Health and research ethics.

*Conflict of interest statement.* None declared.

## Annexe

### One selected and partly abbreviated health systems-associated target for each SDG

#### 1. End poverty in all its forms everywhere

Reduce at least by half the proportion of men, women and children of all ages living in poverty in all its dimensions according to national definitions.

#### 2. End hunger, achieve food security and improved nutrition and promote sustainable agriculture

End hunger and ensure access by all people, in particular the poor and people in vulnerable situations, including infants, to safe, nutritious and sufficient food all year round.

#### 3. Ensure healthy lives and promote well-being for all at all ages

Achieve universal health coverage, including financial risk protection, access to quality essential healthcare services and access to safe, effective, quality and affordable essential medicines and vaccines for all.

#### 4. Ensure inclusive and equitable quality education and promote lifelong learning opportunities for all

Ensure that all girls and boys complete free, equitable and quality primary and secondary education leading to relevant and effective learning outcomes.

#### 5. Achieve gender equality and empower all women and girls

End all forms of discrimination against all women and girls everywhere.

#### 6. Ensure availability and sustainable management of water and sanitation for all

Achieve access to adequate and equitable sanitation and hygiene for all and end open defecation, paying special attention to the needs of women and girls and those in vulnerable situations.

#### 7. Ensure access to affordable, reliable, sustainable and modern energy for all

Ensure universal access to affordable, reliable and modern energy services.

#### 8. Promote sustained, inclusive and sustainable economic growth, full and productive employment and decent work for all

Achieve full and productive employment and decent work for all women and men, including for young people and persons with disabilities, and equal pay for work of equal value.

#### 9. Build resilient infrastructure, promote inclusive and sustainable industrialization and foster innovation

Develop quality, reliable, sustainable and resilient infrastructure, including regional and transborder infrastructure, to support economic development and human well-being, with a focus on affordable and equitable access for all

#### 10. Reduce inequality within and among countries

Empower and promote the social, economic and political inclusion of all, irrespective of age, sex, disability, race, ethnicity, origin, religion or economic or other status.

#### 11. Make cities and human settlements inclusive, safe, resilient and sustainable

Ensure access for all to adequate, safe and affordable housing and basic services and upgrade slums.

#### 12. Ensure sustainable consumption and production patterns

Significantly reduce toxic releases to air, water and soil in order to minimize their adverse impacts on human health and the environment.

#### 13. Take urgent action to combat climate change and its impacts

Integrate climate change measures into national policies, strategies and planning.

#### 14. Conserve and sustainably use the oceans, seas and marine resources for sustainable development

Prevent and significantly reduce marine pollution of all kinds, in particular from land-based activities, including marine debris and nutrient pollution.

#### 15. Protect, restore and promote sustainable use of terrestrial ecosystems, sustainably manage forests, combat desertification, and halt and reverse land degradation and halt biodiversity loss

Integrate ecosystem and biodiversity values into national and local planning, development processes, poverty reduction strategies and accounts.

#### 16. Promote peaceful and inclusive societies for sustainable development, provide access to justice for all and build effective, accountable and inclusive institutions at all levels

Develop effective, accountable and transparent institutions at all levels and ensure responsive, inclusive, participatory and representative decision-making at all levels.

#### 17. Strengthen the means of implementation and revitalize the global partnership for sustainable development

Respect each country’s policy space and leadership to establish and implement policies for poverty eradication and sustainable development.
