# A systems perspective on the importance of global health strategy developments for accomplishing today’s Sustainable Development Goals

**DOI:** 10.1093/heapol/czz124

**Published:** 2019-09-26

**Authors:** Jens Byskov, Stephen Maluka, Bruno Marchal, Elizabeth H Shayo, Astrid Blystad, Salome Bukachi, Joseph M Zulu, Charles Michelo, Anna-Karin Hurtig, Paul Bloch

*Health Policy and Planning*, doi: 10.1093/heapol/czz042.

In the original version of the above paper, Stephen Maluka's name was presented as ‘Stephen A Maluka’ in error. This has now been corrected online and in print.

